# Recruitment and retention in a multicentre randomised controlled trial in Bell's palsy: A case study

**DOI:** 10.1186/1471-2288-7-15

**Published:** 2007-03-28

**Authors:** Brian McKinstry, Victoria Hammersley, Fergus Daly, Frank Sullivan

**Affiliations:** 1 Community Health Sciences, University of Edinburgh, 20 W Richmond Street, Edinburgh, UK; 2 Community Health Sciences, University of Dundee, Mackenzie Building, Kirsty Semple Way, Dundee, UK

## Abstract

**Background:**

It is notoriously difficult to recruit patients to randomised controlled trials in primary care. This is particularly true when the disease process under investigation occurs relatively infrequently and must be investigated during a brief time window.

Bell's palsy, an acute unilateral paralysis of the facial nerve is just such a relatively rare condition. In this case study we describe the organisational issues presented in setting up a large randomised controlled trial of the management of Bell's palsy across primary and secondary care in Scotland and how we managed to successfully recruit and retain patients presenting in the community.

**Methods:**

Where possible we used existing evidence on recruitment strategies to maximise recruitment and retention. We consider that the key issues in the success of this study were; the fact that the research was seen as clinically important by the clinicians who had initial responsibility for recruitment; employing an experienced trial co-ordinator and dedicated researchers willing to recruit participants seven days per week and to visit them at home at a time convenient to them, hence reducing missed patients and ensuring they were retained in the study; national visibility and repeated publicity at a local level delivered by locally based principal investigators well known to their primary care community; encouraging recruitment by payment to practices and reducing the workload of the referring doctors by providing immediate access to specialist care; good collaboration between primary and secondary care and basing local investigators in the otolarnygology trial centres

**Results:**

Although the recruitment rate did not meet our initial expectations, enhanced retention meant that we exceeded our planned target of recruiting 550 patients within the planned time-scale.

**Conclusion:**

While difficult, recruitment to and retention within multi-centre trials from primary care can be successfully achieved through the application of the best available evidence, establishing good relationships with practices, minimising the workload of those involved in recruitment and offering enhanced care to all participants. Primary care trialists should describe their experiences of the methods used to persuade patients to participate in their trials when publishing their results.

## Background

It is notoriously difficult to recruit patients to randomised controlled trials in primary care [1]. This is particularly true when the disease process under investigation occurs relatively infrequently and must be investigated during a brief time window.

Bell's palsy, an acute unilateral paralysis of the facial nerve is just such a relatively rare condition. It affects 25–35 people per 100 000 in the population per annum, most commonly in the age group 30–45 years [2]. So, on average every year a general practitioner will only see one or two patients who have developed the condition. Although most recover well, 30% of patients have a poor recovery with continuing facial disfigurement, psychological difficulties and sometimes facial pain. There has been conflicting evidence of the efficacy of the use of steroids [3] and anti viral agents [4], but there have been indications that either or both may be effective if started within 48 hours of onset. Our trial sought to determine if treatment with prednisolone and aciclovir (separately or in combination) administered in the first 72 hours of the onset of symptoms was more cost effective than placebo in hastening the resolution of the illness and in preventing long term sequelae. The primary end point was incomplete recovery of facial motor function (grade III) as measured by the House-Brackmann scale [5] at 9 months. (We used the House-Brackmann scale as it was the facial nerve grading system accepted by the American Academy of Otolaryngology/Head and Neck Surgery in the United States as the standard used in reporting results at the time we designed the study). Normally primary care and accident and emergency department staff are the only health professionals to see patients early enough to recruit them to such a study and only a co-ordinated approach across a large population would provide sufficient numbers to allow a satisfactory power to be achieved. In addition we thought it advisable that confirmatory examination by a second doctor should take be undertaken. As trial drugs had to be held securely, centrally this was undertaken in secondary care by otolaryngology specialist registrars or consultants in 14 centres in Scotland.

In this case study we describe the organisational issues presented in setting up a large randomised controlled trial of the management of Bell's palsy across primary and secondary care in Scotland and how we managed to successfully recruit and retain patients presenting in the community.

### Study Design

In summary the study design was a 2^2 ^factorial randomized controlled trial [6] offering aciclovir or steroids or both or placebo.

Patients identified in primary medical and dental care or Accident and Emergency (A&E) departments and those who approached NHS24 (a 24 hour medical advice line in Scotland similar to NHS Direct in England and Wales which also co-ordinates all general practice out-of-hours consultations) with an appropriate description of symptoms were asked to attend a local otolaryngology specialist as an emergency for examination, full explanation of the trial, randomised and given therapy. Randomisation was undertaken securely by robotic telephone randomisation controlled by the Health Services Research Unit in Aberdeen [7]. Patients would then be visited by a researcher in their home at baseline (between 3 and five days after onset) and 3 months. If resolution had not occurred a third visit would take place at 9 months. At each visit the researcher would take four digital photographs of the patient for subsequent rating by the House-Brackmann scale, and patients were asked to complete a variety of questionnaire scales exploring health, pain and perceived appearance [8-10].

### Deciding on sample size

The literature on treatment effects is sparse and contradictory. We considered three different scenarios which would provide 80% power at the 5% significance level (2-sided) to detect 10%, 12% and 15% difference between active and placebo treatments (assuming no interaction between treatments). These scenarios required recruitment and successful retention of 656, 470 and 314 patients respectively.

The annual incidence of Bell's palsy in Scotland was estimated at 33 per 100,000 a year using information derived from the Scottish Practice Team Information Continuous Morbidity Recording, a figure roughly in keeping with recent international estimates [11]. This project collects data on presenting illness routinely from 75 Scottish general practices known to be broadly representative of the population as a whole [12]. Given the eligible population of 4.3 million for Scotland the expected number per year in Scotland would be 1420 after one year, and 2129 over 18 months. Due to early delays in obtaining permission from all sites across Scotland the recruitment period was extended to two years.

We recognised that not all of these patients would be notified or recruited. It was important therefore to pilot notification of the condition prior to running the study to determine if general practitioners considered the condition to be of significant importance to become involved in a trial and what proportion of patients would be recruited. In order to test this we piloted a notification in process in one region of Scotland. With the co-operation of the local research networks in Tayside and Fife we asked general practitioners to notify us of all patients presenting with Bell's palsy over a period of one month. As a result of this exercise we determined that we would be able to recruit 1/3 of those presenting within 48 hours of diagnosis. Of those we assumed that 2/3 would remain in the trial for review at 9 months. In order therefore to recruit and retain the 470 patients necessary to detect a 12% difference in treatment effect from Scottish recruitment we needed to recruit continuously for 18 months.

## Methods

### Recruitment

To have any prospect of recruiting one third of all presentations of Bell's palsy in Scotland all general practitioners, accident and emergency departments and unscheduled care services had to be involved. In addition regional otolaryngology services were required. As it was considered possible that some patients might present through their dentists we initially hoped to include this group in recruitment. However, in the end recruitment was restricted to three dental hospital sites and they contributed only one participant.

### Keeping it simple

As it was unlikely that individual general practitioners or accident and emergency doctors would be involved more than once in the trial it was essential that their role should be clearly delineated and relatively simple to carry out and that instruction should be available relatively easily. Recruiting doctors' involvement was restricted to diagnosis followed by determining the patients' interest in participating, excluding patients who were not eligible and a telephone referral to the on-call otolaryngology doctor. The trial process actually constituted a reduction in workload for most general practitioners who would normally be expected to follow-up patients. The trial also offered immediate access to specialist assessment which would not be provided under normal care. Both of these attributes were found to be very attractive to general practitioners during the planning phase of the trial.

It was essential that all hospital otolaryngology staff to whom the referrals would be made (generally junior staff working both during the day and out-of hours) knew about the trial and their role in it and that any briefing was regularly repeated to ensure that new members of staff were informed. Prior to the start of recruitment otolaryngology staff were briefed during visits from regional research associates; thereafter departments (where necessary) were updated six-monthly, immediately after the twice-yearly redistribution of staff in otolaryngology departments.

### Publicising the trial

Doctors need to be reminded regularly of an ongoing trial of a condition which occurs relatively sporadically [13]. In addition, there is a high turnover among staff in accident and emergency departments and training grades in general practice and otolaryngology. A variety of strategies publicising the trial were set in motion.

#### Mail-shots

The responsibility for keeping doctors informed about the on-going trial was taken on by SPPIRe [14] (Scottish Professionals and Practices Interested in Research) the Scottish national primary care research framework. All general practitioners in the four participating regions of Scotland were sent a mail-shot outlining the trial and explaining how to take part. We emphasised the importance of the condition and the simplicity of involvement. The mail-shots were in colour designed to be attractive and, based on evidence from the literature, we highlighted the benefits to patients [15] and remuneration to general practitioners [16] for taking part and letters were signed by well know local general practitioner 'champions' [17]. Separate mail-shots went out to non-principal doctors and registrars. The trial was also highlighted in Local Medical Committee briefings to general practitioners. We estimate each quarterly mail-shot took about a day of researcher time in each of the four participating regions

A&E departments were kept informed by literature and posters from the centre; similarly general practice co-ops through literature and posters distributed with SPPIRe's help and NHS 24 by direct contact with the study centre. We found that the most attention-grabbing poster was one showing photographs of a patient at onset. Every mail-shot included the project's web address.

#### Project Website

This had a simple web address [17], was clear and easy to navigate, with instructions on how to take part in the trial and was regularly updated with information on the progress of the trial. The site was easily found with simple Google terms.

#### Media

In order to heighten and maintain the profile of the study we contacted professional magazines, national press and radio. We were fortunate that a medical graduate and former sufferer who regularly works in a variety of media, Graeme Garden, offered to speak to media colleagues on our behalf to provide his insight into the condition. In all we had two professional magazines, several newspaper articles, a radio programme and a media website dealing with the topic during recruitment. We took advantage of two BMJ articles on Bell's palsy to respond with details of the study. All of these activities may have helped to keep the study in the eye of our target group for recruitment. Such activities did take several days in terms of planning, writing and interviews but could reasonably be fitted in around the general working of the project.

#### Educational meetings

We took every opportunity to raise awareness and build the profile of the study including conference presentations and workshops. However, these exercises connected with relatively few recruiting general practitioners and emergency room staff, and it is hard to know what impact if any they had on recruitment.

#### Regular feedback on the trial

In the quarterly mailings to general practitioners organised by the SPPIRe nodes we took the opportunity both to let them know that the study was still ongoing and the current recruiting status.

#### Remuneration

Following negotiation with primary care Research and Development departments, general practices were offered £51 for recruiting patients into the trial and for any ongoing explanation and care that might be required. In rare cases this fee was intended additionally to cover the situation where a patient preferred to use their general practitioner surgery rather than their home for the researcher's visits.

#### Out-of-hours recruitment and follow-up

With a condition as alarming as Bell's Palsy, it is likely that sufferers will seek attention soon after onset so we anticipated that presentations were likely to occur out of normal working hours. Also treatment had to be started within a relatively short time window. It was essential therefore to have a system to capture this. Additionally as many sufferers would quickly return to work follow-ups were scheduled at convenient times. The research team was therefore prepared to both recruit and follow-up cases outside of normal working hours. Of the cases that were recruited 28% randomised outside normal working hours (5% NHS24, 7% accident and emergency department, 16% out of hours general practitioner). With regard to subsequent follow-ups 10% were carried out at weekends and 22% were carried out before 8.30 AM or after 6.30 PM on weekdays.

## Results

### Problems

In keeping with the experience of many researchers [19,20], the process of obtaining approvals from primary and secondary care research and development committees requiring a wide variety of different types of application followed by requests for honorary contract status for the researchers was long and frustrating. We had estimated this would take three months, but in fact it took seven.

### Recruitment and retention

**Figure 1:**
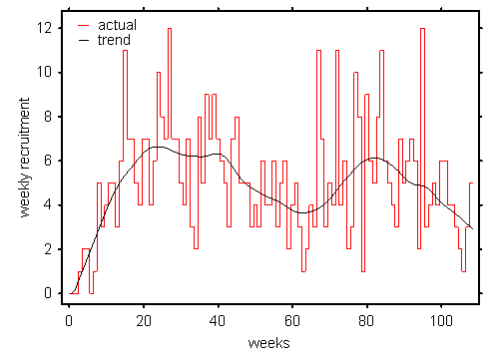
Weekly recruitment figures showing the underlying recruitment rate.

**Figure 2:**
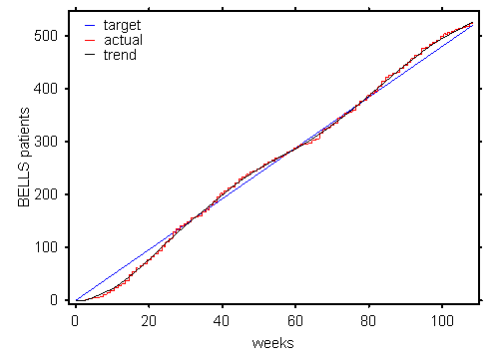
Retention and target recruitment.

We had calculated that, based on our expected retention rate, we would require around ten recruits per week in order to achieve our recruitment and retention target of 480 in 18 months. Initial recruitment took place in summer and was very slow. We were recruiting around six per week. We were unsure if this was because we had miscalculated the actual incidence or if we had been failing to recruit incident cases. We wondered about the possibility of a seasonal effect and there is some literature to suggest that may have been true [21]. Certainly there was a subsequent dip the following summer also. However, as the study progressed it became clear, that retention exceeded expectation and that in fact only six recruits per week was needed to hit target (see figure 1). By week 40 however, we started to become optimistic that we would meet our target (see figure 2).

Forty-four patients were excluded because they were notified more than 72 hours after onset, the other main reason for exclusion being diabetes. Both of these reasons constitute a failure of the recruiters to suitably screen potential participants, however, we thought it better to have inappropriate referrals that could subsequently be excluded (all patients were examined and treated) than risk discouraging recruiters from sending appropriate patients. In 36 cases other patients who were referred for inclusion in the study were lost for a variety of other reasons mainly due to wrong diagnosis (n = 13) or administrative problems with the referral process or at the trial site (n = 15).

There was some variation in recruitment across the study sites as may be expected, with the lead site recruiting substantially in relation to expected prevalence than the other the other three, possibly due heightened awareness in the local general practitioner population (see table 1). The impact of a move of the otolaryngology centre from a city centre location to a peripheral hospital caused a marked reduction in recruitment in one of the centres during the last 6 months of the project, suggesting that convenience of access to the trial site is an important factor in recruitment.

**Table 1:** Cases by region

**Region**	**Number successfully recruited**	**Number known missed**	**Total cases known**	**% of total known case recruited**	**Number of cases expected (population based)**	**% of expected population recruited**
Grampian & Highland	109	26	135	81	360	**23**
Tayside & Fife	110	59	169	65	365	**30**
Lothian & Borders	88	40	128	69	385	**23**
Glasgow & the West	244	85	329	74	1090	**22**

**Total**	**551**	**210**	**761**	**72**	**2200**	**25**

### Retention

Thirty-two people were lost to follow-up because they found the trial too intrusive (n = 8), were unhappy with their randomisation to active (n = 4) or inactive treatment (n = 6), could not be contacted (n = 9), died (not-treatment related) (n = 3), or because of exclusions found after randomisation (n = 2).

.

## Discussion

Although we were ultimately successful in recruiting and retaining sufficient study subjects, there were several lessons to derive from the study, some of which bear out earlier papers which have highlighted engagement and organisational issues [13]. By paying close attention to existing literature in the planning phase and the difficulties experienced early in the recruitment phase we were able to identify barriers to participation.

We consider that there were several key issues which we felt contributed to the success of this study:

### The research addressed a clinically important research question

Our pilot work suggested that Bell's palsy was a topic in which general practitioners were interested, felt there was no clear guidance on management and thought it was a condition worthy of research. Evidence in the literature for the influence of the importance of research topic on recruitment is mixed. A review by Rendell and colleagues [22] identified two studies considering this issue and neither study found a significant link between research topic and recruitment. Conversely, Prescott and colleagues [23] found two studies that presented modest evidence for improved recruitment in trials addressing clinically important questions. However, in the absence of strong evidence to the contrary it makes logical sense to conclude that research questions should be important enough for clinicians to be comfortable with taking part.

### A trial co-ordinator and researchers willing to recruit and follow up patients seven days a week

A large proportion of patients were recruited (28%) and followed up (32%) outwith normal working hours. We felt that willingness to provide this option undoubtedly improved both recruitment (particularly given the tight time window) and retention and is probably the main reason for the trial's success. Many patients made it plain they could not take time off work for the study. Without this recruitment would undoubtedly have taken considerably longer. Researchers were informed at the time of recruitment that he project required evening and weekend work. There were no additional costs in terms of overtime. Researchers had the potential to reclaim out of hours work as "time in lieu".

### National visibility and repeated publicity at a local level delivered by locally based principal investigators well known to their primary care community

We are not sure how much national publicity contributed to the success of the study. For a relatively rare condition such as Bell's palsy, keeping general practitioners informed about the study was essential. Providing regular feedback on the progress of the study with simple graphics on eye-catching paper was a method which kept the study in general practitioners' minds without seeming like a continuous barrage of advertisements. This has also been found to be important by others [15]. Repeating this information particularly around the time of changes of junior staff was considered particularly important. The literature emphasised the benefits to patients of taking part, a factor known to be important to recruiting doctors [15]. Additionally the use of locally known and trusted opinion leaders was considered likely to boost recruitment. The evidence in the literature for this is somewhat sparse. Although there is some evidence linking the use of respected opinion makers with the adoption of guidelines [18], there is contradictory evidence that there is a similar impact on recruitment to trials. De Wit and colleagues [24] explored reasons for recruitment to a trial on the management of dyspepsia and found that doctors who stated that personal knowledge of the researchers was a reason for participation were less likely than others to recruit patients, however, those who stated that the participation of a respected academic research group in the process was a motive for recruiting were more likely to recruit. Borgiel and colleagues [25], in a quality of care study in Canada, however, found that personal knowledge of a researcher was predictive of participation in research in primary care.

### Payment to practices for recruitment

Our initial discussion with doctors suggested that payment was important. However, the literature around payment for recruitment is contradictory. De Wit [24] found that doctors who rated financial payments as an important incentive were no more likely to recruit to a trial than those who did not (the incentives in the trial were very modest) and Silagy [26] found that financial incentives were the least important influence in decisions to take part in a study in an Australian survey. In contrast to this, cash has been found to be an incentive improving questionnaire returns from general practitioners [16] and other survey based research has suggested that financial compensation is an important However, some have expressed concerns that payment may adversely influence informed consent in recruiting to trials [27].

### Reducing workload for the recruiting doctors

Workload has been considered to be an important factor in recruitment to trials in several studies [15,28]. In our trial the workload for the doctors was probably less than it would have been for normal care. Additionally general practitioners had the advantage of being able to refer their patients for specialist assessment very quickly which meant they could offer a positive reason to patients to potentially become involved in the trial whilst easing the burden of recruitment.

### Local investigators in local sites

Basing local investigators in the otolaryngology trial centres ensured that junior staff were regularly reminded about the study, and having a large number of secondary care centres throughout Scotland meant that patients had less distance to travel. The sudden drop in referrals to one centre that had relocated less conveniently during the course of the trial underlines the importance of this feature.

We believe this method of locally based recruitment in secondary and primary care co-ordinated centrally by SPPIRe, Scotland's national primary care research framework, has been found to be particularly successful. Further similar large scale studies are planned.

## Conclusion

While difficult, recruitment to and retention within multi-centre trials from primary care can be successfully achieved through the application of the best available evidence, establishing good relationships with practices, minimising the workload of those involved in recruitment and offering enhanced care to all participants. However, good quality research into effective recruitment to trials is sparse and there is a need for a more comprehensive evidence base. Primary care trialists should describe their experiences of the methods used to persuade patients to participate in their trials when publishing their results.

## Competing interests

The author(s) declare that they have no competing interests.

## Authors' contributions

BM planned and wrote the paper, FD co-ordinated the Bell's palsy trial and helped plan and write the paper, VH helped plan and write the paper, FS led the Bell's palsy trial and helped plan and write the paper. All authors read and approved the final manuscript.

## Pre-publication history

The pre-publication history for this paper can be accessed here:


